# Improving Diagnostic Accuracy of Dermoscopically Equivocal Pink Cutaneous Lesions with Reflectance Confocal Microscopy in Telemedicine Settings: Double Reader Concordance Evaluation of 316 Cases

**DOI:** 10.1371/journal.pone.0162495

**Published:** 2016-09-08

**Authors:** J. Łudzik, A. M. Witkowski, I. Roterman-Konieczna, S. Bassoli, F. Farnetani, G. Pellacani

**Affiliations:** 1 Department of Dermatology, University of Modena and Reggio Emilia, Modena, Italy; 2 Department of Biostatistics and Telemedicine, Jagiellonian University Medical College, Krakow, Poland; University of Queensland Diamantina Institute, AUSTRALIA

## Abstract

**Background:**

Solitary pink lesions in differential diagnosis with hypopigmented/amelanotic melanoma present a diagnostic challenge in daily practice and are regularly referred for second expert opinion. Reflectance confocal microscopy (RCM) has been shown to improve diagnostic accuracy of dermoscopically equivocal pink lesions. No studies have been performed to evaluate the effect of adding a second expert reader and automatic removal of lesions with discordant management recommendations and its potential effect on diagnostic sensitivity and final management of these lesions in retrospective or telemedicine settings.

**Objective:**

To improve diagnostic accuracy and reduce potential mismanagement of dermoscopically equivocal pink cutaneous lesions by implementing double reader concordance evaluation of RCM images.

**Methods:**

316 dermoscopically equivocal pink lesions with dermoscopy-RCM image sets were evaluated retrospectively. Accuracy of three readers was evaluated by single reader evaluation of dermoscopy only and dermoscopy-RCM image sets and finally by double reader evaluation of dermoscopy-RCM image sets. Lesions with discordant diagnosis between two readers were automatically recommended for excision.

**Results:**

Dermoscopy only evaluation resulted in an overall sensitivity of 95.9% and specificity of 33.6%, with 1 of 12 amelanotic melanomas mismanaged. Dermoscopy-RCM image set single reader evaluation resulted in an overall sensitivity of 93.9% and overall specificity of 54.2%, with 1 of 12 melanomas mismanaged. Dermoscopy-RCM image set double reader concordance evaluation resulted in an overall sensitivity of 98.3% and specificity of 42.7%, with no amelanotic melanoma mismanagement.

**Conclusion:**

Evaluation of dermoscopy-RCM image sets of equivocal pink lesions by a single reader in telemedicine settings is limited by the potential for misdiagnosis of dangerous malignant lesions. Double reader concordance evaluation with automatic referral of lesions for removal in the case of discordant diagnosis improves the diagnostic sensitivity in this subset of lesions and reduce potential misdiagnosis in settings where a second expert opinion may be employed.

## Introduction

Solitary pink cutaneous lesions in differential diagnosis with hypopigmented/amelanotic melanoma (AMM) may present a diagnostic challenge in daily practice due to their diverse clinical and dermoscopic presentation and often times paucity of diagnostic clues. Pink lesion differential diagnosis ranges from inflammatory processes to aggressive skin malignances that may present as pink to red papules, plaques or nodules with little to absent melanin pigmentation [1,2]. The use of dermoscopy increases the sensitivity and specificity of diagnosis as it enables the user to visualize minimal pigment deposition as well as specific vascular patterns of the lesions which may not be detectable with the naked eye [3–6]. Pizzichetta et al. demonstrated that dermoscopy had higher sensitivity and specificity (89% and 96%) than clinical diagnosis (65% and 88%) and Menzies et al. reported lower sensitivity for amelanotic/hypomelanotic nodular melanoma (84%) [7–8]. Recently, the implementation of reflectance confocal microscopy (RCM) has become well-received in the dermatology community as a supplementary non-invasive tool that can further improve early diagnosis of skin tumors at the patient bedside by providing an *in-vivo* optical biopsy at histologic resolution down to a depth of 200 μm of skin tissue [9]. In addition to the improvement of diagnostic accuracy, RCM has been shown to significantly reduce the number of unnecessary excisions in different settings and be suitable for application in pink lesions [10–16]. Recently the reliability of tele-consultation with RCM images has been tested in an observational retrospective setting which showed the capability of an accurate diagnosis but with risk of mismanagement, where diagnostic accuracy depends greatly on the level of expertise [17].

No specific studies have been performed to evaluate the potential effect of double reader concordance evaluation of dermoscopy-RCM image sets to improve accuracy and safety of management of dermoscopically equivocal pink lesions. Our goal in this study was to determine potential improvement of diagnostic accuracy and chosen management of this group of lesions in a telemedicine consultation setting applying double reader concordance evaluation to reduce potential equivocal lesion management mistakes in telemedicine settings where a second expert opinion may be employed.

## Materials and Methods

### Patient Population

This was an Ethical Committee approved observational retrospective analysis within the European Project DIAGNOPTICS (grant n. 621066) based on 316 consecutive dermoscopically equivocal pink cutaneous lesions collected from 316 different patients that were evaluated with dermoscopy and RCM imaging for diagnostic decision in order to rule out a diagnosis of melanoma during the period of January 2009 to January 2012 at the Dermatology Department at the University of Modena and Reggio Emilia (UNIMORE). All research was approved by IRB (University of Modena and Reggio Emilia IRB: Comitato Etico Provinciale di Modena; Jagiellonian University Collegium Medicum IRB: Komisja Bioetyczna Uniwersytetu Jagiellonskiego) and was conducted according to the principles expressed in the Declaration of Helsinki. Patient consent was obtained in written form. All lesions were evaluated and imaged with digital dermoscopy and with RCM, followed by excision for histopathological analysis. The inclusion criteria for lesions in this study were: (i) lesion detected by clinical naked-eye examination with absent pigmentation or containing less than 10% pigment; (ii) absence of pigment network; (iii) all lesions excised with matching histopathology report; (iv) availability of digital dermoscopy images; (v) availability of a complete standard set of RCM images. We tested the accuracy of an experienced dermoscopy reader (Reader 1) who evaluated only dermoscopy images via telemedicine cloud based server versus two separate experienced RCM readers who individually evaluated dermoscopy-RCM image sets, Reader 2 at UNIMORE and Reader 3 via telemedicine cloud based server. All readers were blind to histopathology diagnosis. Department patient code and private information (age, sex, lesion body site location and history) was not provided to the readers and the image sets were placed into separate computer folders and numbered (001 to 316) differently than the department patient code to ensure that the evaluations were made in blind.

### Imaging Protocol and Evaluation

Digital dermoscopy images were obtained with DermLite FOTO System (DermLite Photo 3Gen-San Juan Capistrano, CA, USA). RCM images were obtained with a reflectance confocal microscope (Vivascope 1500; MAVIG GmbH, Munich, Germany) using a 830 nm laser at a maximum power of 20 mW. RCM images of 0.5 mm x 0.5 mm were acquired with a lateral resolution of 1 μm and an axial resolution of 3–5 μm and stitched into composite images that covered between 4 to 8 square mm mosaics (VivaCube; Caliber I.D., Inc., Rochester, NY, USA). A minimum of three mosaics were obtained at different depths, corresponding to the stratum granulosum/spinosum, the dermal-epidermal junction, and the papillary dermis. The cases in this study included the following malignancies: amelanotic/hypomelanotic melanomas (AMM), basal cell carcinomas (BCC), squamous cell carcinomas (SCC) and benign lesions (including naevi, solar lentigo (SL), seborrheic keratosis (SK)), or other benign lesions, and were made available for the evaluation during the time period of January to March 2016. The images were evaluated in blind by 3 readers each with at least two years of dermoscopy and RCM clinical experience. Image sets for Reader 1 and Reader 3 were made accessible on a DICOM and HIPAA compliant secure cloud based server where access to data was only possible with individual login and password. Reader 2 retrospectively evaluated the patient folders at UNIMORE. Each reader was asked to provide their management decision and suspected diagnosis based only on the provided image sets into a Microsoft excel file. Management was grouped into two categories: (i) excision or (ii) no-excision. Management decision confidence level was graded: (i) low or (ii) high. In order to test concordance of double reading, data from the excel files (Reader 2 and Reader 3) were matched and chosen for automatic management with excision when (1) management decision was concordant for excision, (2) management decision was discordant or (3) management decision was concordant for benign diagnosis but with double low confidence.

### Statistical Analysis

Statistical evaluation was carried out with SPSS statistical package (IBM, Armonk, NY, U.S.A.). Diagnostic values of sensitivity and specificity of individual readers were calculated for malignant versus benign lesions. Absolute and relative frequencies of confidence in benign and malignant lesion management were calculated. χ² test was used to compare confidence level of management with actual histopathologic diagnosis. Cohen’s kappa coefficient was calculated in order to find association between double reader management and histologic diagnosis, a P-value < 0.05 was considered significant. Receiver Operating Characteristic (ROC) was calculated using binary management values (0: all benign lesion types, 1: all malignant lesion types) as the state variable and the overall management decision with confidence level (1: excision, high confidence; 2: excision, low confidence; 3: no-excision, low confidence; 4: no-excision, high confidence) as the test variable.

## Results

The study population consisted of 172 histopathology proven malignant cases including AMM (12 cases, 3.8%), BCC (138 cases, 43.7%), SCC (20 cases, 6.3%) and two other malignancy types which included one Kaposi sarcoma and one syringoid eccrine carcinoma (2 cases, 0.6%). The remaining 144 cases included nevi (64 total cases, 20.3%; of which 8 were spitz naevi), SK/SL/LPLK/AK (33 cases, 10.4%) and other benign lesions including dermatofibromas, angiokeratomas and angiomas classified as other (47 cases, 14.9%). The evaluations were performed by Reader 1 (dermoscopy only) and by Readers 2 and 3 (dermoscopy-RCM image sets) for a total of 316 dermoscopy evaluations and 632 dermoscopy-RCM image set evaluations. Reader 1 (dermoscopy only) achieved an overall sensitivity of 95.9% and specificity of 33.6%, with 1 of 12 (8.3%) melanomas misdiagnosed.

Dermoscopy-RCM image set single reader evaluation resulted in an overall sensitivity of 93.9% (ranging between 91.3% to 96.5%) and specificity of 54.2% (ranging between 53.1% to 55.2%). Sensitivity of AMM diagnosis was 91.7% for Reader 2 and 100% for Reader 3 respectively, with 1 out of 12 MMs (8.3%) mismanaged by Reader 2. Overall diagnostic sensitivity for BCC was 94.2% (ranging between 90.6% to 97.8%) and 100% for SCC. Overall specificity of nevi diagnosis was 57.1% (ranging between 55.3% to 58.9%), 42.5% for SK/SL/LPLK/AK (ranging between 36.4% to 48.5%) and 63.9% (ranging between 61.7% to 66.0%) for other benign lesions. ROC area under the curve for Reader 2 was 0.776 (p < 0.001) and 0.812 for Reader 3 (p < 0.001).

Dermoscopy-RCM double reader evaluation utilizing the method of computer automated referral of lesions for removal when two readers had discordant management decisions resulted in an overall sensitivity of 98.3% and specificity of 42.7%; lesions with concordant management decisions for no-excision were considered benign and those with concordant management decisions for excision were considered malignant (Table 1). Combined AMM diagnostic sensitivity for both readers was 100%, with no mismanagement of AMM. Overall diagnostic sensitivity for BCC was 98.6% and 100% for SCC. Overall specificity of nevi diagnosis was 40.6%, 27.3% for SK/SL/LPLK/AK, and 57.4% for other benign lesions (Table 2). The overall concordance between both readers was high (kappa 0.08, p = 0.072).

**Table 1:** Overall diagnostic performance of all readers.

	Dermoscopy ONLY Reader 1	RCM Reader 2	RCM Reader 3	Combined RCM (Reader 2 and 3)
**Overall Sensitivity**	166/173 (95.9%)	158/173 (91.3%)	167/173 (96.5%)	170/173 (98.3%)
**Overall Specificity**	48/143 (33.6%)	79/143 (55.2%)	76/143 (53.1%)	61/143 (42.7%)
**AMM Sensitivity**	11/12 (91.7%)	11/12 (91.7%)	12/12 (100%)	12/12 (100%)
**BCC Sensitivity**	132/138 (95.7%)	125/138 (90.6%)	135/138 (97.8%)	136/138 (98.6%)
**Benign Nevi (no Spitz) Specificity**	20/56 (35.7%)	31/56 (55.4%)	33/56 (58.9%)	25/56 (44.6%)

AMM: amelanotic/hypomelanotic melanoma, BCC: basal cell carcinoma, RCM: reflectance confocal microscopy.

**Table 2:** Double reader management.

Management	AMM	BCC	SCC	Other Malignant	Naevi	Spitz	SK/SL/LPLK/AK	Other Benign	Total
**NO-Excision**	0	2	0	1	25	1	9	27	65
**Excision**	12	136	20	1	31	7	24	20	251
**Total**	12	138	20	2	56	8	33	47	316

AMM: amelanotic/hypomelanotic melanoma, BCC: basal cell carcinoma, SCC: squamous cell carcinoma, SK: seborrheic keratosis, SL: solar lentigo, LPLK: lichen planus-like keratosis, AK: actinic keratosis.

Individual reader confidence was evaluated for Readers 2 (R2) and 3 (R3). For overall malignant lesions R2 managed 143/172 cases (83.1%) and R3 managed 156/172 cases (90.7%) with high confidence. For overall benign lesions R2 managed 54/144 cases (37.5%) and R3 managed 61/144 cases (42.0%) with high confidence. For malignancies present in the study population, high confidence for AMM diagnosis was reported in 7/12 cases (58.3%) by R2 and 11/12 cases (91.7%) by R3. BCC diagnosis with high confidence was reported in 115/138 cases (83.3%) by R2 and 128/138 cases (92.8%) by R3. SCC diagnosis with high confidence was reported 100% of cases by R2 and 17/20 cases (85%) by R3.

**Fig 1:**
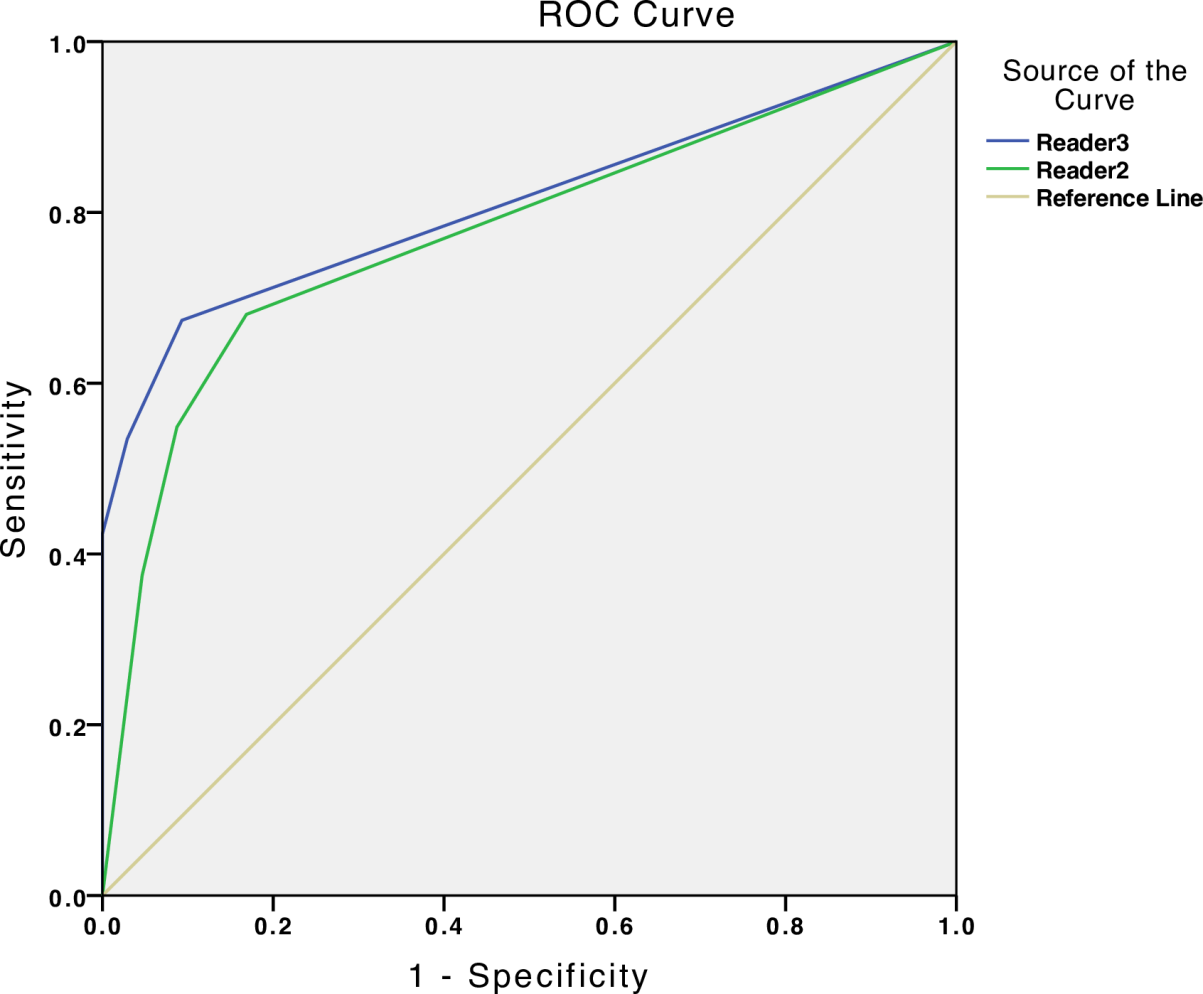
ROC curve: Reader 2 versus Reader 3 (dermoscopy-RCM image sets).

For benign lesions present in the study population, high confidence for naevi diagnosis was reported in 20/56 cases (35.7%) by R2 and 28/56 cases (50.0%) by R3. Spitz naevi diagnosis with high confidence was reported in 2/8 cases (25.0%) by R2 and 1/8 (12.5%) by R3. SK/SL/LPLK/AK diagnosis with high confidence was reported in 13/33 (39.4%) by R2 and 10/33 (30.3%) by R3. For all other benign lesions R2 reported high confidence in diagnosis in 19/47 cases (40.4%) and R3 22/47 (46.8%). R2 and R3 confidence level frequencies are reported in **(Table 3)** and **(Table 4)** (Fig 1).

**Table 3:** Confidence level frequencies (Reader 2 dermoscopy-RCM imaging).

	AMM	BCC	SCC	Other Malignant	All othe Naevi	SpitzNaevi	SK/SL/LPLK/AK	Other Benign	Total
**E-HC**	7	115	20	1	16	5	14	11	189
**E-LC**	4	10	0	0	9	0	3	7	33
**NE-LC**	1	5	0	1	11	1	3	10	32
**NE-HC**	0	8	0	0	20	2	13	19	62
**Total**	12	138	20	2	56	8	33	47	316

E-HC: excision with high confidence, E-LC: excision with low confidence, NE-LC: no-excision with low confidence, NE-HC: no-excision with high confidence, AMM: amelanotic/hypomelanotic melanoma, BCC: basal cell carcinoma, SCC: squamous cell carcinoma, SK: seborrheic keratosis, SL: solar lentigo, LPLK: lichen planus-like keratosis, AK: actinic keratosis.

**Table 4:** Confidence level frequencies (Reader 3 –dermoscopy/RCM imaging).

	AMM	BCC	SCC	Other Malignant	All other Naevi	Spitz Naevi	SK/SL/LPLK/AK	OtherBenign	Total
**E-HC**	11	128	17	0	13	7	16	11	203
**E-LC**	1	7	3	0	10	0	5	5	31
**NE-LC**	0	3	0	2	5	0	2	9	21
**NE-HC**	0	0	0	0	28	1	10	22	61
Total	12	138	20	2	56	8	33	47	316

E-HC: excision with high confidence, E-LC: excision with low confidence, NE-LC: no-excision with low confidence, NE-HC: no-excision with high confidence, AMM: amelanotic/hypomelanotic melanoma, BCC: basal cell carcinoma, SCC: squamous cell carcinoma, SK: seborrheic keratosis, SL: solar lentigo, LPLK: lichen planus-like keratosis, AK: actinic keratosis.

## Discussion

The purpose of our study was to determine the management safety of equivocal pink cutaneous lesions referred for telemedicine second expert opinion based on dermoscopy-RCM image sets. Since pink lesions present fewer discernable features than their counterparts these lesions are more often referred to colleagues for second opinion or excised at once due to uncertainty. If a clinician excises all equivocal lesions sensitivity will approach 100% but comes at the expense of a significantly decreased specificity and imbalance between patient safety and healthcare expenditures. Our goal in this study was to test a variety of methods of store and forward retrospective evaluation of patient cases and if the addition of a second blind confocal reader has the capability to produce safer management results of equivocal lesions, particularly in telemedicine settings.

In the general population AMM accounts for up to 8% of cutaneous melanomas and represents an important diagnostic pitfall for clinicians. AMM also presents a surgical management problem as it can mimic a variety of benign and malignant lesions such as BCC, AK, benign inflammatory plaques and Paget or Bowen disease [14,15]. The dermoscopic algorithms that are routinely used for pigmented lesions are not as helpful in diagnosing these non-pigmented tumors and while presence of atypical vessels seen under dermoscopy may be suggestive of a particular diagnosis unfortunately these criteria are rarely specific [2,4]. Since AMM diagnosis are usually delayed and present in more advanced forms it is important to utilize the safest method of evaluation of these tumors and their counterparts in settings where an expert second opinion may be required and most often acquired through telemedicine.

In our study Reader 1 evaluated only digital dermoscopy images via telemedicine access which resulted in an overall sensitivity of 95.9% (BCC: 95.7% and MM: 91.7%) and a specificity of 33.6%, with mismanagement of 1 AMM and 16 BCCs (based on 632 total evaluations). In a real-world clinical scenario the mismanagement of an AMM can be potentially life threatening since in settings lacking strict follow up control with sequential digital dermoscopy these lesions may go unnoticed for an extended period of time. Our results delineate the limitation of single reader image evaluation specifically in the case of equivocal pink cutaneous lesions where the diagnostic sensitivity may be insufficient to keep the percentage of missed melanomas at a safe level due to the absence of typical criteria in this group of lesions and lower diagnostic confidence level [18–22].

RCM has been shown to improve diagnostic accuracy of pink lesions in various settings thanks to the refractive index of melanocytic structures that produce visible reflection in RCM images regardless of their clinical lack of pigmentation [14,15]. A recent study showed that sensitivity is higher for experienced RCM users versus those who are new to the field (91.0% vs. 84.8%), but that specificity is quite similar (80.0% vs. 77.9%) [13]. In our study single reader dermoscopy-RCM image set evaluation was comparable with recently published literature showing that RCM has a high overall sensitivity for pink cutaneous lesions (R2: 91.3%; R3: 96.5%) [10–12,14,15,23]. An individual category sensitivity for AMM (R2: 91.7%; R3:100%), BCC (R2: 90.6%; R3: 97.8%) and SCC (R2/R3: 100%) was obtained by both readers. High sensitivity for SCC detection may be an effect of training confocal readers to remove suspicious lesions presenting with significant dyskeratosis and accompanying atypical vasculature, typically found in Bowens disease and/or SCC. Specificity for pink naevi excluding spitz naevi (R2: 55.3%; R3: 58.9%) showed significant improvement over dermoscopy only evaluation (35.7%) verifying that RCM permits visualization of morphologic features providing more cellular information for the reader and therefore the potential to improve both diagnostic accuracy and confidence in this group of equivocal lesions. In the SK/SL/LPLK/AK category specificities (R2: 48.4%; R3: 36.4%) were acceptable and also in concordance with literature [10–13]. The low specificity for recognizing spitz naevi (R2: 25.0%; R3: 12.5%) confirms previously reported limitations of RCM application in this subset of lesions [23].

Regarding confidence level in diagnosing AMM R2 managed 7/12 (58.3%) correctly with high confidence, 4/12 (33.3%) correctly with low confidence and mismanaged 1/12 (8.3%), reporting the mismanaged lesion as benign with low confidence. R3 managed 11/12 (91.7%) correctly with high confidence and 1/12 (8.3%) correctly with low confidence. The variability of confidence between two readers with similar experience levels suggests that this group of lesions may contain subtle morphologic changes may not be as pronounced as in pigmented MM counterparts and leave more room for potential mistakes in lesion management.

For BCC diagnosis R2 managed 115/138 (83.3%) correctly with high confidence, 10/138 (7.2%) correctly with low confidence and mismanaged 5/138 (3.6%) with low confidence and 8/138 (5.8%) with high confidence. R3 managed 128/138 (92.8%) correctly with high confidence 7/138 (5.1%) with low confidence and mismanaged only 3/138 (2.2%) with benign diagnosis and low confidence. These values show that RCM is valid for confident BCC diagnosis even in the case of equivocal pink lesions. The BCC cases mismanaged by both readers were all early superficial types present only on the trunk or legs and away from cosmetically sensitive areas such as the face. Since BCC rarely metastasis these mismanaged false negative lesions would not present significant health risk to these patients and most likely would be diagnosed on a future follow-up visit when dermoscopic features would become mature and more apparent.

In our study double reading of dermoscopy-RCM image sets enhanced the overall diagnostic sensitivity (98.3%) and minimized the likelihood to mismanage an invasive melanoma that was otherwise mismanaged with dermoscopy only and dermoscopy-RCM image set single reader evaluations. This previously mismanaged AMM was selected for removal per double reader evaluation due to reader discordance in selected management (R2: no-excision; R3: excision). Additionally, BCC sensitivity (98.6%) was also improved with double reading evaluation reducing 16 mismanaged BCC by single reader evaluation (based on 632 evaluations) to only 2 mismanaged cases. 12/16 of these cases (75.0%) were selected for removal due to reader discordance and 2 cases (12.5%) were removed based on concordant benign diagnosis with both readers reporting low confidence. Overall the improvement of sensitivity resulting for the addition of a second evaluator benefited most from discordant management decision rather than two readers reporting benign management decision with double low confidence. Our results further delineate dermoscopy and RCM as complimentary/synergistic methods for diagnosis of amelanotic/lightly-colored skin lesions as delineated in a recent study by Guitera et al. where RCM sensitivity was 66.6% and 72.9% for melanoma and BCC diagnosis, respectively and its specificity for non-malignant lesion diagnosis was 56.1%. Moreover, RCM is the only clinically applicable tool permitting clear visualization of morphologic features of non-pigmented pink cutaneous lesions that can be applied to both melanocytic and non-melanocytic lesions at the bedside and in a telemedicine setting [24–28].

In settings where sequential digital dermoscopy (videodermoscopy) is available we recommend to follow any negative flat lesion that has been referred for confocal teleconsultation and evaluated as benign with no-excision management as videodermoscopy permits objectively controlled follow-up of these lesions referred for consultation and the possibility to identify changes in the lesions over time that can be signs of early malignancy. In the case of any uncertainty in raised or palpable lesions biopsy is advised to reduce the risk of potential mismanagement of a false negative diagnosis/management recommendation [28,29]. Our study may have been limited by the use of patient dermoscopy-RCM image transfer in a telemedicine setting where full clinical and dermoscopic view as well as complete history of the patient was absent resulting in decreased management confidence level. In the future an important consideration to improve this limitation could be to implement patient mole-mapping integration with telemedicine in order to provide a complete set of patient data for the reader including full clinical and dermoscopic view of multiple moles on the patient.

Dermoscopically equivocal pink lesions fall into a category of difficult to diagnose lesions that more commonly invoke the need for second expert opinion that is now possible through telemedicine store and forward technology [30,31]. Distant evaluation of equivocal skin lesions using digital dermoscopy images only in telemedicine settings may not provide enough information to make a safe diagnosis with high sensitivity while maintaining an equitable specificity [20–22]. Additionally, combined dermoscopy-RCM image evaluation of pink lesions at a distance is also limited due to the possibility to miss small clues in the image mosaics that can influence the diagnostic decision and confidence [14,15,17].

In conclusion, we showed that the application of a second expert check system in telemedicine settings with an automatic management decision to excise any lesions with discordant management between two readers considerably improved the sensitivity threshold of diagnosis and safety for the patient. Additionally, the use of RCM that permits near histologic non-invasive biopsy enabled the maintenance of an acceptable specificity of lesion diagnosis. With the broadened popularity of RCM and its diffusion into clinical practice the use of telemedicine evaluation will inevitably increase in order to connect patients with experts around the globe particularly in equivocal case presentations. The addition of double reader concordance evaluation of dermoscopy-RCM image sets in this group of lesions may be considered as a safety net for continued spread of the technology and safe management of equivocal pink lesions. Our study should be considered as preliminary and additional studies are warranted to confirm these results.
