# Correction: Improving Diagnostic Accuracy of Dermoscopically Equivocal Pink Cutaneous Lesions with Reflectance Confocal Microscopy in Telemedicine Settings: Double Reader Concordance Evaluation of 316 Cases

**DOI:** 10.1371/journal.pone.0340311

**Published:** 2026-01-02

**Authors:** J. Łudzik, A. M. Witkowski, I. Roterman-Konieczna, S. Bassoli, F. Farnetani, G. Pellacani

The second affiliation for the first author is incorrect. The correct affiliation is not indicated. J. Łudzik is not affiliated with #2 but with: Jagiellonian University Medical College, Department of Bioinformatics and Telemedicine, Kraków, Poland.

The corresponding author’s email address is incorrect. A. M. Witkowsk’s email address is: aw@medvice.health.
